# The Role of Congestion-Adaptive Routing Protocol in Mobile Ad Hoc Networks

**DOI:** 10.1155/tswj/6296169

**Published:** 2025-08-10

**Authors:** Vijay Uttam Rathod, Shyamrao Vasantrao Gumaste, Himadri Majumder

**Affiliations:** ^1^Department of Computer Engineering, MET's Institute of Engineering, Nashik, Maharashtra, India; ^2^Department of Mechanical Engineering, G H Raisoni College of Engineering and Management, Pune, Maharashtra, India

**Keywords:** AODV, CRP, DSR, MANETs, OLSR, routing congestion, TORA

## Abstract

In MANETs, the congestion-adaptive routing protocol (CRP) enhances data delivery by dynamically adjusting routes based on real-time congestion, unlike traditional protocols that lack congestion adaptability. The present paper discusses the CRP proposed protocols for evaluating the E2E delay, PDR, overhead, and energy consumption across various data rates (1–40) and communication times (0–900 ms) using the ns-3 simulator. The obtained results were compared with the DSR, TORA, OLSR, and AODV protocols. Results indicate that CRP delivers superior performance to DSR, TORA, OLSR, and AODV in congestion-aware conditions. The optimal improvements in the CRP protocol are observed for an E2E delay of 85% and a PDR of 53.84%. In the case of routing overhead and energy consumption, the performance of the CRP protocol has been reduced by 20.68% and 23.07%, respectively, compared to the DSR and AODV protocols.

## 1. Introduction

Mobile ad hoc networks (MANETs) represent self-organizing and self-configuring multihop wireless networks that operate without centralized control, where wireless connections and spontaneous interactions occur between mobile nodes in a highly dynamic manner [1]. In the last decade, MANETs have had wide applications in disaster relief situations, military communications, meetings, emergencies, sensor networks, and in many other temporary scenarios where no physical infrastructure exists or is difficult to establish. However, due to the dynamic topology and lack of central administration, MANETs have limitations and inherent risk factors, such as frequent topology changes causing link breaks, delays, and congestion [2]. Numerous routing protocols for MANETs have been presented as solutions to these problems. To classify them, use different criteria, such as proactive against on-request or single-route versus multipath routing. Proactive protocols create pathways between each pair of nodes in advance, even when no transmission is necessary [3]. Another criterion is whether routing is congestion-adaptive or congestion-non-adaptive in MANETs. The current routing protocols fall under the category of congestion-non-adaptive routing. In this work, we propose a new routing protocol that falls within the category of congestion-adaptive routing protocol (CRP). The existing routing protocols include congestion control, congestion awareness, and nonadaptive congestion control, which are not adaptive. When congestion occurs in the primary route of the network, CRP adaptively adjusts the traffic flow [4, 5]. The major issue observed in MANETs is packet loss, which is caused by channel errors, node mobility, and congestion at the network layer. Routing allows congestion to happen, which is detected and handled by congestion control. Using congestion-non-adaptive routing to manage congestion leads to longer delays, increased overhead, and significant packet losses. Hence, we need an advanced routing protocol, such as CRP, to overcome the issues of packet loss mentioned above. Extensive literature studies were reported by researchers worldwide. Kanthimathi and JhansiRani [5] have considered Modified Ad hoc on-demand Distance Vector (MAODV) Protocol in wireless ad hoc networks to offer an optimal routing-centered congestion control scheme used in descent deep learning neural networks and levy flight-based black widow optimization. The optimization technique is used to explore the data transmission performance in terms of residual energy, path lifetime, distance, and link cost. The study's output shows that MAODV was enhanced in considered wireless networks, improving energy consumption, end-to-end delay (E2E), and packet delivery ratio (PDR) by 2.62 J, 0.019 ms, and 96.9%, respectively, at 100 nodes. Gayatri and Kumaran [6] proposed a least common multipath-based routing and fuzzy logic propagation to enhance routing efficiency and minimize data traffic, based on the multipath source and target nodes in MANETs. And hypothetical analysis shows that the adopted multipath routing has enhanced efficiency by 96.7% in load balancing for MANETs. Similarly, Pa and Mandala [7] proposed a novel technique to enhance routing efficiency using ant colony optimization (ACO) and the new congestion avoidance method (NCAM) in wireless mesh networks (WMNs). Moreover, the researcher has employed three methodologies, including the discovery of congestion at every ideal node, updating values, and moving data packets, to prepare a suboptimal path. The problem was simulated in ns-2, and it was observed that NCAM shows lower packet loss, superior network throughput, and a decrease in E2E compared to the ACO method. Tilwari et al. [8] conducted a study on E2E, energy cost, convergence time, and data packet loss ratio in MANETs using a Q-learning algorithm that incorporates mobility, residual energy, and link quality-aware multipath routing (MRLAM) techniques. The obtained results were compared with those of the multipath optimized link state routing (MP-OLSR) protocol and MP-OLSRv2 schemes, respectively. And it seems that using the MRLAM technique end-to-end, the delay was reduced from 15% to 10%, energy consumption from 33% to 23%, convergence time from 16.49% to 11.34%, and data packet loss ratio from 30.76% to 24.59%, compared to other well-established schemes. Hong et al. [9] employed game theory and deep reinforcement learning (DRL) in MANETs to enhance the balance between network capabilities and arbitrary topologies. The results were obtained using the optimization method, and it was found that the network delay and residual energy variance decreased, while the network lifetime increased. The GDR model achieves a 10.5% higher E2E compared to well-known techniques. Suresh et al. [10] worked on the optimal route selection (ORS) of the cluster technique in MANETs using an energy-efficient multicast routing protocol to verify the quality of service (QoS) in networks. The output of work conducted by the author shows that the proposed ORS MANETs have lesser jitter in channel and network latency and higher PDR and throughput, respectively, compared to well-known techniques. Bhardwaj and El-Ocla [11] perform an optimization analysis to solve congestion problems in MANETs using the combined routing protocol, ad hoc on-demand multipath distance vector with fitness function (AOMDV-FFn), and further integrate the protocol with the genetic algorithm (AOMDV-GA). The analysis reveals that the efficient route of TCP congestion control enhancement for random loss is achieved with AOMDV-FFn compared to the AOMDV-GA technique. Sadreddini et al. [12] introduced a novel routing protocol for intelligent reflecting surfaces (IRSs) in MANET (MRIRS) to analyze the performance of PDR, E2E, throughput, and link failure. Furthermore, the author simulated the program on 40 topologies and three destination pairs for the same source in MRIRS, obtaining an 8% and 800 ms enhancement in PDR and E2E, respectively. Additionally, it was observed that, with an average node speed of 8 m/s, the improvement in throughput achieved by MRIRS is 114.88 kbps, despite a 15.97% link failure rate. At a node speed of 16 m/s, 24% of link failures were observed, which is equivalent to 98% for the remaining protocols. Tran et al. [13] numerically study the performance of queuing and routing delay, PDR, overhead, and energy consumption using an innovative deep reinforcement learning-based quality-of-service routing (DRQR) protocol in cognitive radio MANETs. Hence, the results obtained by the DRQR protocol improved by 84% for Ad hoc On-Demand Distance Vector (AODV) compared to other vectors. Safari et al. [14] conducted a fuzzy-based numerical study in MANETs using the Cross-Layer Adaptive Fuzzy-based Ad hoc On-Demand Distance Vector Routing Protocol (CLAF-AODV) to analyze the stability, QoS, and adaptability of broadcast traffic. To minimize network broadcast traffic, the authors have considered the standard AODV and fixed probability AODV (FP-AODV) algorithms in the context of fuzzy logic. The prominent results were obtained in broadcast packet reduction and advanced network performance for FP-AODV compared to standard AODV. Khudayer et al. [15] perform simulation work using six routing protocol mechanisms, including source routing dynamic source routing (DSR), zone-based DSR, reliable DSR, and segment-based DSR, which are standardized routing mechanisms predefined by the protocol. The authors' proposed mechanism is a zone-based route discovery mechanism (ZRDM) and a link failure prediction mechanism (LFPM) to study the performance of PDR and routing load. Using the ns-3 simulator, the results were evaluated for six mechanisms, and it was found that the most prominent results were obtained for the proposed ZRDM and LFPM mechanisms compared to the standard ones.

## 2. Congestion-Adaptive Routing

For MANETs, the CRP is a unicast routing protocol [16, 17]. In CRP, when packets pass through the primary route, congestion occurs at any node along that route. The previous node in the primary route will get alerts from the congested node [18, 19]. In such a situation, the last node on the same route will function to create an alternate route, known as a bypass route, to minimize network congestion [20, 21].

The following components are included in CRP, which is provided on demand:

A. Monitoring the network congestion
B. Identifying the primary path for data transmission
C. Bypass route
D. Efficiently balance network traffic with adaptive congestion handling
E. Optimization of multipath
F. Recovery from network failures

To understand the concept of “bypass,” an example has been explored. The “bypass” concept has been adopted due to the nonavailability of this scenario in existing routing protocols, and it has been extensively discussed for each component.

### 2.1. Example Based on “Bypass” Creation

**Figure 1:**
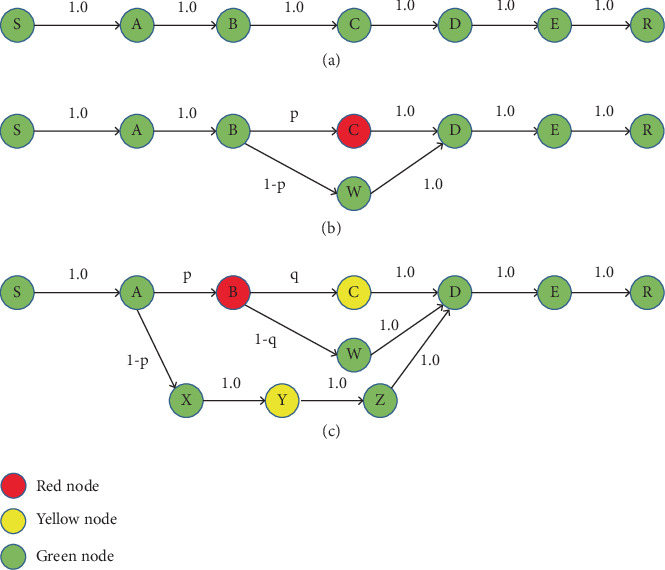
Network routing scenarios under three conditions: (a) without congestion, (b) with congestion at a single node, and (c) with congestion affecting multiple nodes.

Figure 1 illustrates a bypass concept in CRP with an example. The figure is explained using different color nodes, such as red, yellow, and green, which represent areas that are very congested, likely to be congested, and far away from congestion, respectively, where S and R represent the source and destination nodes and intermediate nodes are A, B, C, D, E, and W to Z. In Figure 1a, the route S to R is known as the primary route. Moreover, every packet follows the primary route. In the exact figure, Node C experiences network congestion after a certain amount of time has passed. In such a situation, Node C warns the neighboring node regarding the congestion. This congestion is illustrated in Figure 1b, where Node B searches for the next noncongested node, which is Node D, immediately after Node C. Here, the network traffic coming to Node B→C is diverted to Node D via Node B→W→D with the help of the bypass route having the Probabilities p and 1-p, respectively, as shown in Figure 1b. Similar congestion occurred in Node B (as shown in Figure 1c). The congestion will be warned to Node A, and the network traffic link from A to B will be split through a new bypass route: A→X→Y→Z→D. To avoid congestion in routing, the bypass concept is an efficient route. The significant concerns are not addressed in this example and require further exploration in the following subsection.

#### 2.1.1. Monitoring the Network Congestion

When the number of packets arriving at a node exceeds the buffer capacity, congestion occurs, causing the node to drop excess packets. To monitor this congestion, various metrics can be used, including packet loss due to buffer overflow, the average and standard deviation of packet delays, the queue length, and the frequency of late or retransmitted packets. The buffer capacity of the node, represented by “*r*”, is determined by Equation (1).

(1) $$\begin{array}{c} r=\frac{\text{number of packets buffered}}{\text{buffer size}}. \end{array}$$

A node is considered “green” (indicating low congestion) when *r* ≤ 1/2, meaning the buffer is less than 50% full. It is classified as “yellow” (potentially congested) when *r* ∈ 1/2, indicating the buffer is more than half full but not filled. Finally, the node is marked “red” (congested) when *r* ∈ 3/4, 1, showing that the buffer is nearly or full.

#### 2.1.2. Identifying the Primary Path for Data Transmission

**Figure 2:**
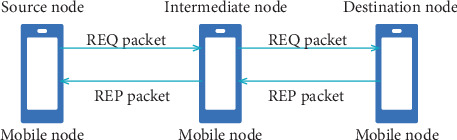
Schematic diagram of primary route discovery in CRP.

In Figure 2, the path from the source to the destination node (and vice versa) is referred to as the primary route, and nodes along this path are called primary or intermediate nodes [22]. The primary and bypass tables are the two routing tables that each node owns, denoted as “prTab” and “brTab,” respectively. When straight packets are on the primary path, prTab is used; otherwise, brTab is used for bypass routes. The different abbreviations are used in the first-level routing table as shown in Table 1.

Two strategies were applied in CRP when congestion occurs in the network, necessitating a reduction in the route's burden [23]. In the first case, if a “red” congestion condition arrives at a node, then REQ must be dropped. In the second case, continuing from the first case, the same REQ is received by Receiver R, which reaches Node N, where the same node already has the presence of R due to the prior connection that has been established. In such a situation, Node N needs to pass REQ to the next hop as prTab [N, R].hop to avoid broadcasting and reduce network congestion. Moreover, Node N adds a new entry when the REP is introduced by Receiver R; otherwise, it removes the old entry in the prTab. After this case, there is no data response for R or prTab [N, R]; the entry of R is deleted.

#### 2.1.3. Bypass Route

**Figure 3:**
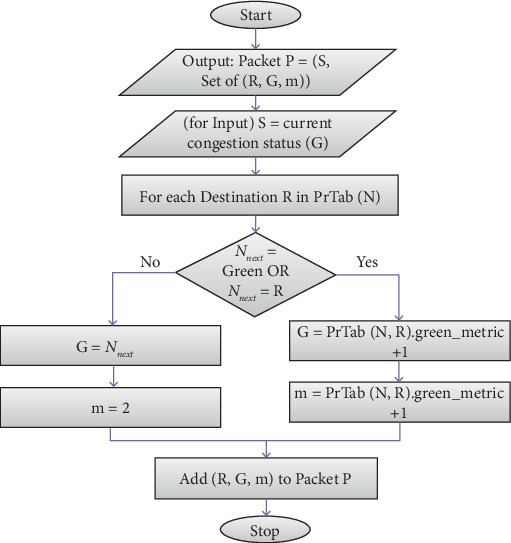
Flowchart for creating UDT.

Periodically, a primary node sends an update packet (UDT) with a TTL value of 1, as illustrated in Figure 3.

This UDT packet includes a set of tuples for every destination listed in the primary routing table, containing the Destination R, the next green Node G, and the distance to that green node mmm. Additionally, the packet carries the current congestion status of the node. Figure 3 shows that the procedure was used to construct the UDT packet.

**Figure 4:**
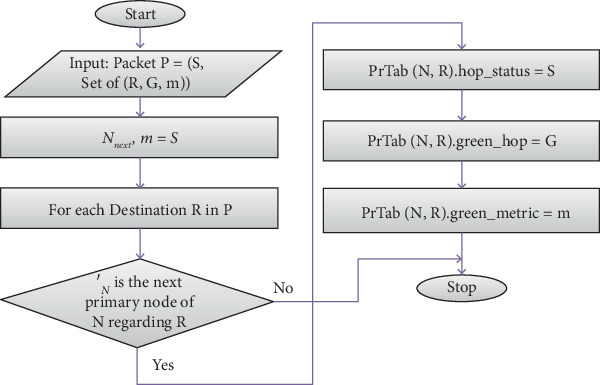
Flowchart for recount.

The goal is for Node N to be alerted to the congestion state of *N*_next_ and understand that the subsequent green node of N is G, which is m hops away on the main path when N gets a UDT packet from its subsequent main node *N*_next_ about target Node R. This knowledge is essential if a bypass is required. The primary table is consequently updated, as seen in the following example of Procedure recvUDT (as shown in Figure 4).

#### 2.1.4. Efficiently Balance Network Traffic With Adaptive Congestion Handling

The Probability p is periodically updated based on the congestion condition at the fresh primary node and the bypass route, in response to congestion caused by network dynamics [24]. The growing status of all bypass nodes determines the congestion condition of a bypass. The bypass route is explained and depicted in Figure 1c. The route is shown from A to X to Y to Z to D in yellow. The basic tenet is that traffic should be enhanced in the primary link if a less congested node leads or drops the primary link. The probability adjustment policy is well presented in Table 2.

**Figure 5:**
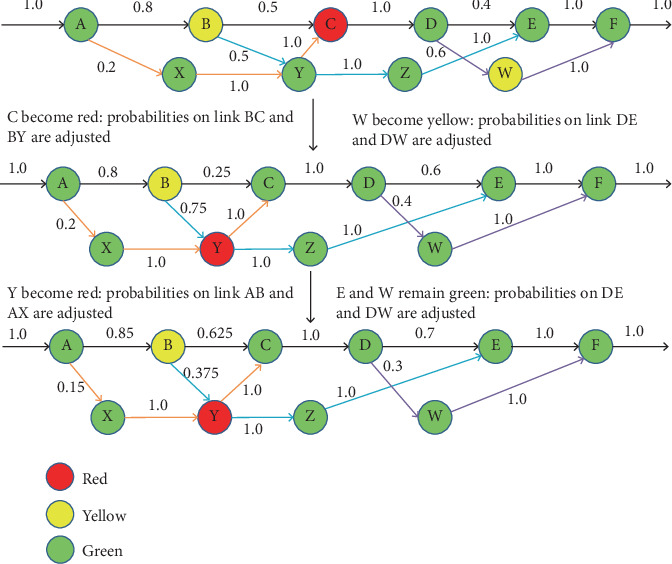
Examples of splitting probabilities that are congestion-adaptive.

Congestion-adaptive routing has been considered for this purpose, as shown in Figure 5. The work has begun with congestion-adaptive routing, where the current bypasses are A→X→Y→C, B→Y→Z→E, and D→W→F. As previously mentioned, C and D are not green, so the bypass starts from B to Destination E. Hence, C turns red, and D becomes green now. When heavy traffic congestion occurs at C, it becomes red and is known to B. Thus, B modifies the probability on the significant Link B→C to 0.5 − 0.5/2 = 0.25 due to the bypass status being green. Similarly, when Node D learns that its bypass has changed to yellow, the probability driven by Link D→E will change to 0.4 + 0.6/3 = 0.6, as shown in Figure 5. Considering the Node Y becomes red and node C and W will remain green, then the new probability is adopted in the Link A→B, B→C, and D→E as 0.8 + 0.2/4 = 0.85, 0.25 + 0.75/2 = 0.625, and 0.6 + 0.4/2 = 0.7, respectively, which can be seen at the bottom of Figure 5.

#### 2.1.5. Optimization of Multipath

**Figure 6:**
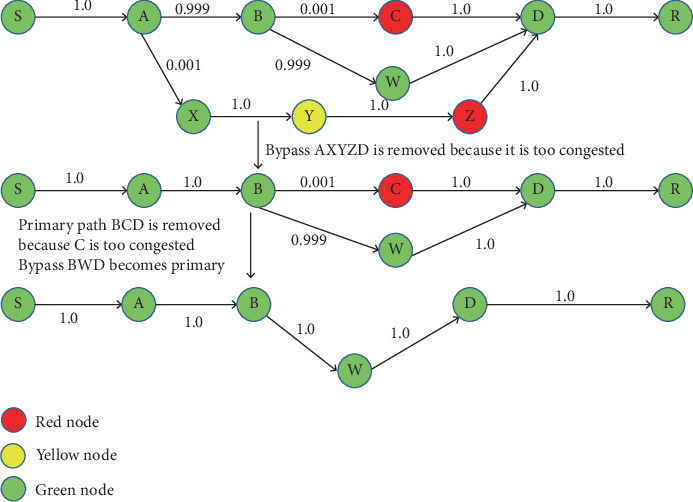
The bypass removal and path switching help manage severe congestion.

In Figure 6, the CRP attempts to utilize fewer multiple pathways to minimize protocol overhead. The PrTab [N, R] prob is approaching 1.0, which means that the bypass or primary route is too congested. In this case, the Link A→X→Y →Z→D bypass route has been removed. Now, in the primary route, the PrTab [N, R] prob approaches zero, which indicates a highly congested node.

In this situation, the main link is detached while the bypass route is made primary. As discussed in the two cases (Figure 6), the routing tables of the bypass nodes are updated in both scenarios, and the changes are communicated to the respective node.

In CRP, a node is only allowed to use one bypass, avoiding the need for multipathing and upholding the protocol's simplicity.

#### 2.1.6. Recovery From Network Failures

**Figure 7:**
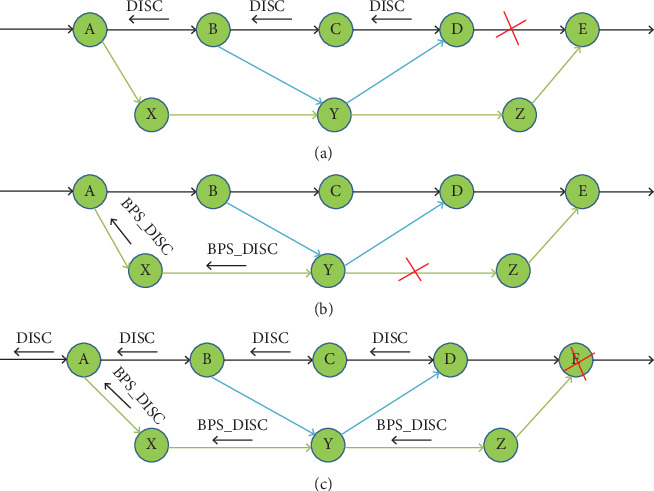
(a) Link DE on primary route fails: primary path ABCDE is removed, bypass BYD is removed, and bypass AXYZE becomes primary. (b) Link YZ on bypass fails: bypass AXYZE is removed. (c) Node E on primary route fails: A forwards DISC upstream (reproduced from Tran and Raghavendra [25]).

After a link failure, a good routing protocol should quickly and gracefully re-establish connectivity. In such a situation, CRP will seek the advantages of the available bypass route. Furthermore, in this section, we will provide an in-depth discussion of three failure cases to understand the concept of failure rehabilitation, with a focus on the single connection from Sender S to Receiver R. In the three cases, the bypass routes are A A→Y→D and A→X→Y →Z→E, as shown in Figure 7.

##### 2.1.6.1. Failure in the Primary Link

A primary connection's starting node, such as Link D to E, transmits a DISC packet upstream along the principal route in the direction of the sender. The DISC noted this visit to the nodes. At Node B, DISC will stop searching for a bypass; however, it appears that this bypass will reach Node E before the Failed Link D→E. Hence, DISC is moved upstream to search for the alternate bypass till Node A. Additionally, it was observed that node A has a bypass that reaches after the Failure Link D→E, that is, at E, and in this way, Sender S finds a fresh primary link to R (as shown in Figure 7a).

##### 2.1.6.2. Failure in the Bypass Link or Node

In Figure 7b, a Bypass Link A→X→Y→Z→E, the Node Y sends BPS_DISC in the upstream in Source Node A direction about the failure, that is, Link Y→Z is failed. Hence, the bypass route is no longer connected.

##### 2.1.6.3. Failure in the Primary Node

This situation combines the two cases mentioned above. When a failure occurs in the primary and bypass nodes, it forwards the DISC and BPS_DISC upstream to Node A, respectively, from Nodes D and Z as in Case 3 of Figure 7c. Meanwhile, the DISC upstream reaches Node A, but it still waits for the BPS_DISC upstream. Once the BPS_DISC upstream reaches Node A, the bypass will be disconnected, and the DISC upstream will still be forwarded on the primary route, as in Case 1 of Figure 7a. And in the event that BPS_DISC upstream does not reach node A, the bypass will act as the primary route, as in Case 2 of Figure 7b. If this delay persists, BPS_DISC is ignored. Such failures in the route are detected and fixed within a short time to ensure the smooth functioning of the network. Tran and Raghavendra [25] provide a detailed explanation of all three potential cases involving failures, including both link failures and node failures, along with illustrative examples for each scenario.

### 2.2. Real-World Implementation or Constraint Scenario

While the CRP has shown promising results in simulation, real-world implementation presents several challenges and considerations. Practical deployment must account for unpredictable environmental factors, hardware limitations, and variability in wireless signal propagation, which are often oversimplified in simulations. Additionally, real-world constraints, such as node mobility, interference, synchronization issues, and energy harvesting limitations, can affect protocol performance. Implementing this protocol would require robust, low-power hardware, and comprehensive testing on physical test beds to validate its adaptability and reliability under dynamic, real-world network conditions.

## 3. Details of Simulation

Table 3 outlines the simulation setup used to evaluate the performance of the CRP in comparison with four established MANET routing protocols: AODV, DSR, Temporally Ordered Routing Algorithm (TORA), and Optimized Link State Routing (OLSR). The evaluation was conducted across fixed data rates of 1, 5, 10, 20, and 40 Mbps. The detailed simulation configuration used to evaluate the performance of the CRP against other MANET routing protocols. The simulation was conducted with 50 mobile nodes deployed in a 1500 × 300 m area. Each node was equipped with a Lucent WaveLAN radio interface operating at a nominal bit rate of 2 Mbps and a radio transmission range of 250 m. The MAC layer followed the IEEE 802.11 distributed coordination function (DCF) standard, and channel behavior was modeled using both the free-space and two-ray ground reflection propagation models. A routing buffer capable of storing 64 data packets was configured, with the maximum node speed set to 4 m per second. Mobility variations were introduced using pause times of 0, 300, 600, and 900 ms, representing different mobility levels from high to no movement. The simulation threshold was set at 0.1, ensuring sensitivity to connectivity and path loss conditions during performance evaluation.

### 3.1. Modeling Energy Consumption and Hardware or Battery Assumptions for Network Nodes

To implement the CRP with energy efficiency in mind, a standard energy consumption model was used, which accounts for transmission, reception, idle, and sleep states. Transmission energy depends on the number of bits and the distance between nodes, modeled as Equations (1) and (2).

(2) $$\begin{array}{c} E_{tx}\left(k,d\right)=E_{\text{elec}.k}+E_{\text{amp}}.k.d^{n}, \end{array}$$

where *k* is the number of bits, *d* is the distance between sender and receiver, *E*_elec_ is the energy consumed by electronics per bit, *E*_amp_ is the energy consumed by the amplifier, and *n* is the path loss exponent (typically 2 ≤ *n* ≤ 4).

While reception energy *E*_*rx*_(*k*) is

(3) $$\begin{array}{c} E_{rx}\left(K\right)=E_{\text{elec}.k}. \end{array}$$

Idle and sleep modes are also considered, with idle consuming moderate power and sleep consuming minimal energy. Assumptions for node hardware typically include low-power microcontrollers (such as the Atmega328) and 802.11-based transceivers with a range of ~100 m. Typical power consumption values are 60 mW for transmission, 50 mW for reception, 45 mW for idle, and less than 1 mW in sleep mode. Each node is assumed to operate on AA batteries with a capacity of 2000 mAh at 3 V, providing approximately 32.4 kJ of total energy. CRP can incorporate these parameters by selecting routes based on both congestion status and residual energy, promoting balanced energy usage and extending overall network lifetime.

### 3.2. Mathematical Relation

#### 3.2.1. PDR

A PDR is the summation of several data packets forwarded from the sender node by the data packet reaching the destination node and shown as Equation (4).

(4) $$\begin{array}{c} \text{PDR}=\frac{\sum P_{d}}{\sum P_{s}}\times 100, \end{array}$$

where *P*_*d*_ and *P*_*s*_ represent the number of data packets sent and delivered, respectively.

#### 3.2.2. Throughput

Throughput is measured using the following expression (Equation 5):

(5) $$\begin{array}{c} \begin{array}{c} G=\frac{\sum B_{r}\times 8}{T}\times 10^{6}, \end{array} \end{array}$$

where *G*, *B*_*r*_, and *T* are the throughput, total number of bytes received, and the simulation time, respectively.

#### 3.2.3. E2E Delay

The total E2E delay is calculated as Equation (6).

(6) $$\begin{array}{c} \begin{array}{c} \text{E}2\text{E}=\frac{\sum_{i=0}^{n}{\text{R}}_{i}-{\text{S}}_{i}}{n} \end{array}, \end{array}$$

where R and S are the receiver and sender, *n* is the number of effectively received packets, and *i* represents the time consumed by the *i*th number of data packets sent and received.

#### 3.2.4. Energy Consumption

The energy consumption (*E*) in joule is measured as Equation (7).

(7) $$\begin{array}{c} \begin{array}{c} E=\overset{m}{\underset{i=0}{\sum }}I_{i}-E_{i}, \end{array} \end{array}$$

where *E* and *I* are the energy consumption and initial energy of the node and *m* is the simulation time.

#### 3.2.5. Routing Overhead (RO) Ratio

The RO ratio is measured as Equation (8).

(8) $$\begin{array}{c} \begin{array}{c} \text{RO}\left(\%\right)=\frac{R_{p}}{R_{p}+D_{p}}\times 100, \end{array} \end{array}$$

where *R*_*p*_ and *D*_*p*_ represent the no. of routing packets and data packets sent, respectively, and *p* represents the data packets.

## 4. Results and Discussions

In our simulation study, CRP was implemented in Network Simulator ns-3 v3.28 using the CMU Monarch wireless extensions and compared against four widely used MANET routing protocols: DSR, TORA, OLSR, and AODV. Performance was assessed under constant data rates of 1, 5, 10, 20, and 40 Mbps. The random waypoint mobility model was employed with a uniform node speed of 4 m/s to simulate movement patterns. To evaluate the effect of mobility, we varied pause times to represent different mobility levels: 0 ms for maximum node mobility and 300, 600, and 900 ms for minimum node mobility. The results derived from these scenarios are presented in the subsequent section.

### 4.1. Highest Node Mobility Scenario (Pause Time: 0 ms)

**Figure 8:**
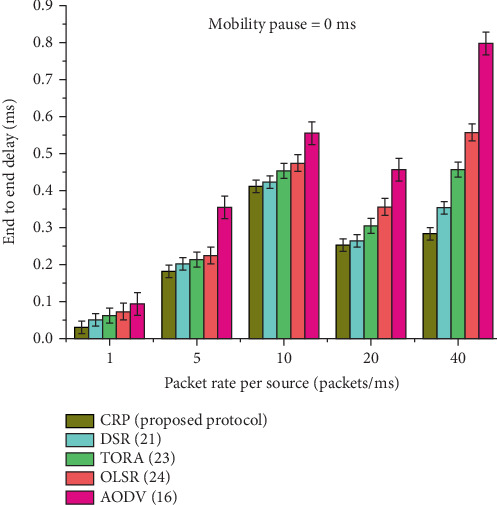
The average E2E delay at a node pause time of 0 ms.

**Figure 9:**
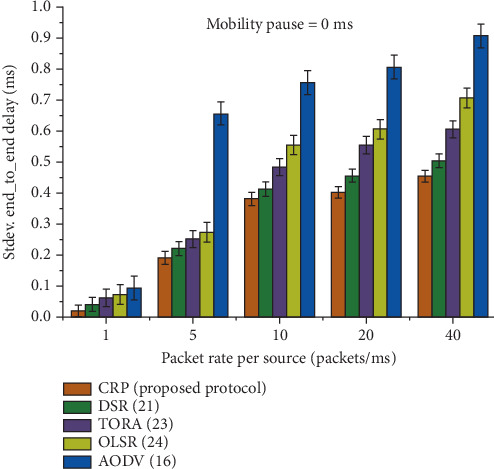
The E2E uniformity delay for a 0 ms pause time.

**Figure 10:**
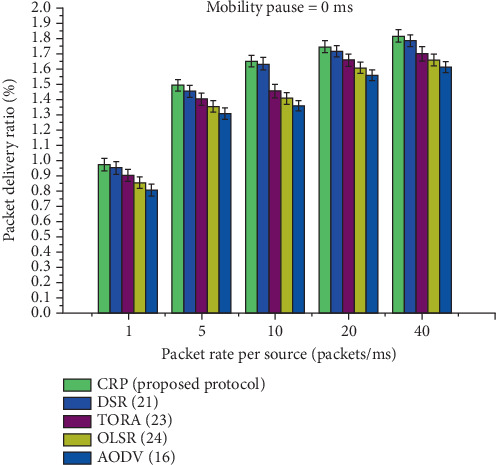
A graphical representation of the PDR for a 0 ms pause time is provided.

**Figure 11:**
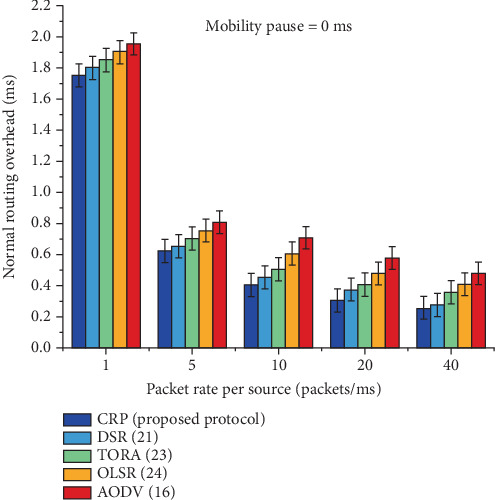
The routing overhead calculation for different protocols when the node pause time is 0 ms.

**Figure 12:**
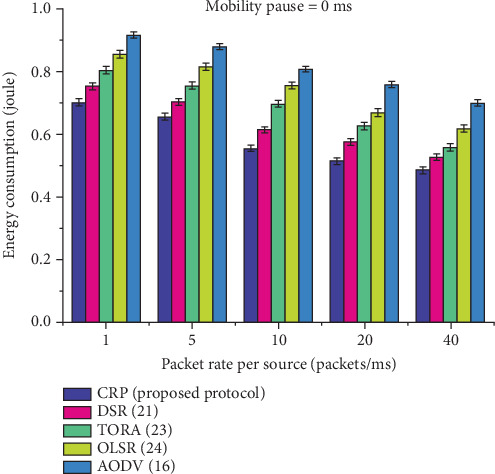
The energy consumption of various protocols at a node pause time of 0 ms.

The maximum node movement was evaluated based on the average E2E delay, PDR, energy consumption, and RO at constant data rates of 1, 5, 10, 20, and 40 Mbps. The results of this evaluation are presented in Figures 8, 9, 10, 11, and 12 and Tables 4, 5, 6, 7, and 8.

It is observed from Table 4 and Figure 8 the E2E delay for different routing protocols, CRP (the proposed protocol), DSR, TORA, OLSR, and AODV, under a node pause time of 0 ms, which simulates highly dynamic network conditions. The delay values are measured at varying data packet transmission rates: 1, 5, 10, 20, and 40 packets per millisecond. Across all rates, the CRP consistently demonstrates lower delay compared to the other protocols. For instance, at a data rate of 1 packet/ms, CRP achieves an E2E delay of 0.030 ms, significantly lower than AODV's 0.094 ms. Even as the rate increases, CRP maintains its performance advantage, achieving delays such as 0.181 s at 5 packets/ms and 0.284 s at 40 packets/ms. The final row quantifies the average performance gain of CRP over the competing protocols, showing reductions in delay ranging from 11.64% to 14.85%, indicating that CRP offers more efficient data transmission in dynamic MANET environments.

CRP dynamically monitors network congestion using real-time feedback from nodes. It detects congestion early and proactively adapts routes, reducing packet queuing and processing delays. CRP's congestion-adaptive nature, efficient bypass routing, and low control overhead lead to significantly reduced delays compared to DSR, TORA, OLSR, and AODV in MANETs. DSR, TORA, OLSR, and AODV lack proactive congestion management, resulting in longer delays during congestion; this has also been observed in previous publications [14].

Table 5 and Figure 9 illustrate the E2E uniformity delay performance of five routing protocols: CRP (the proposed protocol), DSR, TORA, OLSR, and AODV, under the condition of 0 ms node pause time, indicating continuous mobility. The uniformity delay reflects the consistency of delay experienced by packets across various transmission rates: 1, 5, 10, 20, and 40 packets per millisecond. CRP consistently achieves the lowest uniformity delay across all data rates, with values such as 0.021 ms at 1 packet/ms and 0.455 ms at 40 packets/ms, significantly outperforming AODV, which records the highest delays (e.g., 0.095 and 0.909 ms at the same respective rates). As the rate increases, the delays of all protocols increase, but CRP's growth is more moderate, demonstrating better scalability and stability. The percentage gain row highlights CRP's efficiency, showing improvements ranging from 12.71% over DSR to 16.85% over AODV. This indicates that CRP not only maintains low delay but also ensures more uniform and predictable performance in highly mobile environments.

The CRP protocol achieves lower uniformity delay compared to DSR, TORA, OLSR, and AODV primarily due to its efficient route selection and adaptive congestion control mechanisms, which are designed to handle dynamic network conditions with minimal disruption. Unlike traditional protocols that may suffer from frequent route breakages or delayed route discoveries in highly mobile scenarios, CRP proactively predicts channel quality and adjusts routing paths to avoid congested or unstable links. This leads to more stable and consistent packet delivery times across varying data rates. As a result, CRP maintains a lower and more uniform E2E delay, ensuring smoother and more reliable communication, even when node mobility is high and transmission rates increase.

It is investigational from Table 6 and Figure 10, which provides a comparative analysis of the statistical PDR for five routing protocols: CRP, DSR, TORA, OLSR, and AODV, under a 0 ms pause time, indicating continuous node mobility. The PDR values are measured across different transmission rates: 1, 5, 10, 20, and 40 packets per millisecond. CRP consistently demonstrates a higher PDR than the other protocols at all rates, starting at 0.970 at 1 packet/ms and reaching up to 1.814 at 40 packets/ms. In contrast, AODV, which shows the lowest performance, records a PDR of 0.804 and 1.608 at the same respective rates. The increasing trend in PDR for CRP, even under high transmission rates and mobility, indicates its robustness and reliability in delivering packets successfully. The performance gain row shows CRP's advantage over the other protocols, with improvements ranging from 1.76% over DSR to 4.98% over AODV, highlighting CRP's superior capability in maintaining high packet delivery efficiency in dynamic MANET environments.

A CRP enhances packet delivery by maintaining alternative routing paths, ensuring continued data transmission even if the primary route fails. In contrast, DSR, TORA, OLSR, and AODV generally depend on single-path routing without immediate fallback options, making them more vulnerable to disruptions. CRP's integrated congestion awareness, adaptive route adjustment, use of bypass routes, and load balancing contribute to its superior PDR, especially in dynamic and congested MANET conditions. As noted in recent research [11], DSR, TORA, OLSR, and AODV often suffer from repeated packet retransmissions under congestion, further degrading their delivery performance.

It is observed from Table 7 and Figure 11 that the statistical analysis and graphical comparisons of RO for various protocols, including CRP, DSR, TORA, OLSR, and AODV, are presented at a node pause time of 0 ms, representing a highly mobile MANET scenario. RO values are recorded across different data transmission rates: 1, 5, 10, 20, and 40 packets per millisecond. CRP consistently demonstrates the lowest overhead at all rates, starting from 1.751 at 1 packet/ms and decreasing to just 0.255 at 40 packets/ms. In contrast, AODV incurs the highest overhead across all rates, with values ranging from 1.955 to 0.479. The data indicates that as the rate increases, all protocols experience a decrease in overhead, but CRP maintains the most efficient performance. This efficiency is further emphasized by the percentage gain row, which shows CRP's overhead reduction ranges from 6.75% to 9.78% compared to the other protocols. The results highlight CRP's ability to reduce control message exchange and maintain efficient route management in dynamic network conditions.

A CRP effectively reduces RO in MANETs by integrating proactive congestion detection, utilizing bypass routes, and employing localized control strategies. These mechanisms minimize the frequency of control message exchanges, leading to better resource efficiency and overall network performance compared to DSR, TORA, OLSR, and AODV.

Table 8 and Figure 12 detail the energy consumption performance of five routing protocols—CRP, DSR, TORA, OLSR, and AODV—under a 0 ms node pause time, simulating continuous node mobility. The energy usage is measured in joule at transmission rates of 1, 5, 10, 20, and 40 packets/ms. Across all rates, CRP consistently consumes the least energy, starting at 0.702 units at 1 packet/ms and reducing to 0.486 units at 40 packets/ms. In comparison, AODV shows the highest energy consumption, ranging from 0.916 to 0.699 units, indicating less efficiency. The energy savings achieved by CRP become more pronounced at higher data rates, reflecting its design emphasis on energy efficiency through optimized routing, reduced retransmissions, and minimal control overhead. The gain row quantifies CRP's advantage, with energy savings ranging from 9.11% to 12.65% over the other protocols. These results confirm CRP's effectiveness in conserving energy, which is critical for prolonging the operational lifetime of nodes in MANETs.

In MANET environments, CRP consumes significantly less energy than traditional protocols such as DSR, TORA, OLSR, and AODV. This is attributed to CRP's proactive congestion-aware routing, reduced control message exchange, intelligent route management, and even distribution of traffic across the network. These energy-efficient mechanisms not only lower power consumption but also contribute to longer network lifespan and improved system performance. Conversely, DSR, TORA, OLSR, and AODV are more energy-intensive due to their reactive design, limited adaptability to congestion, and higher control overhead, resulting in inefficient route maintenance and increased energy consumption, as documented in [15].

### 4.2. No Node Mobility Scenario (Pause Time: 0 ms)

When the pause period was fixed at 900 ms, mobility did not contribute to network losses; instead, losses were entirely due to channel errors and network congestion. Therefore, we expect CRP to minimize delay and effectively manage network congestion by leveraging bypass paths. The results of the current simulation confirm this expectation.

**Figure 13:**
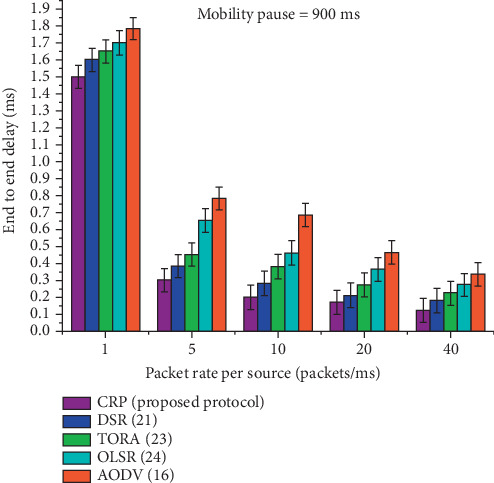
The average E2E delay at 900 ms pause time.

Table 9 and Figure 13 show a comparative analysis of the average E2E delay for five routing protocols—CRP, DSR, TORA, OLSR, and AODV—at a fixed node pause time of 900 ms across varying data rates. The table highlights how CRP consistently achieves the lowest delay at each data rate, starting from 1.500 ms at a rate of 1 and dropping significantly to 0.124 ms at a rate of 40. In contrast, the other protocols exhibit higher delays at every rate, with AODV showing the highest delay values throughout. Notably, as the data rate increases, the delay decreases across all protocols, but CRP maintains a performance edge. The final row shows the percentage gain in delay reduction achieved by CRP compared to the other protocols, with improvements ranging from 15.87% to 18.95%, reinforcing CRP's effectiveness in minimizing delay under the given simulation conditions.

**Figure 14:**
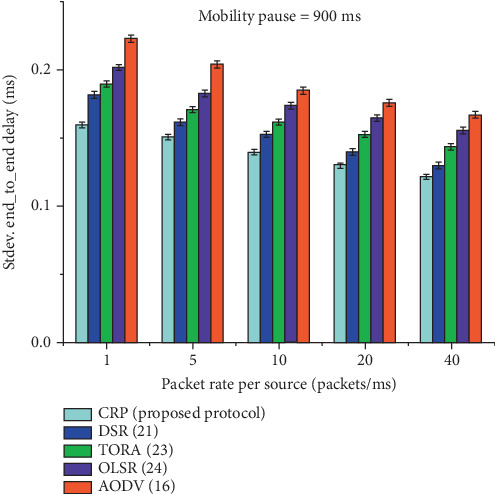
Pictorial representation of E2E delay uniformity at a node pause time of 900 ms.

Table 10 and Figure 14 present the statistical analysis of E2E delay uniformity across different data rates (1, 5, 10, 20, and 40) at a fixed node pause time of 900 ms, comparing the performance of five routing protocols: CRP, DSR, TORA, OLSR, and AODV. The data represent the standard deviation or variability of E2E delay for each protocol, indicating how consistently each protocol handles packet delivery under identical conditions. CRP exhibits the lowest variation in delay across all data rates, starting from 0.160 at rate 1 and steadily decreasing to 0.122 at rate 40, signifying high delay uniformity and stability. Other protocols show higher variability, with AODV consistently having the highest values (0.223–0.167), indicating more erratic delay performance. The percentage gain row quantifies CRP's improvement over each protocol in terms of delay uniformity, ranging from 9.10% to 14.99%. These results statistically reinforce that CRP not only achieves lower average delays but also ensures more predictable and stable performance under varying network loads.

**Figure 15:**
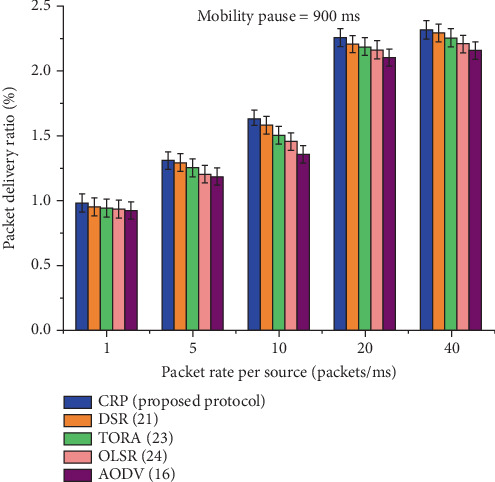
The PDR scheming at a node pause time of 900 ms.

It is seen from Table 11, and Figure 15 presents the statistical data used to evaluate the PDR performance of five routing protocols: CRP, DSR, TORA, OLSR, and AODV, under a fixed node pause time of 900 ms across different data rates. The PDR values for all protocols increase with rising data rates, indicating improved delivery efficiency under heavier traffic conditions. CRP consistently demonstrates superior PDR at every data rate, beginning with 0.980 at a rate of 1 and peaking at 2.314 at a rate of 40. Compared to the other protocols, AODV exhibits the lowest PDR throughout. The percentage gain row at the bottom highlights CRP's performance advantage, showing improvement margins ranging from 1.94% to 4.69% over the other protocols. Statistically, this indicates that CRP not only achieves higher packet delivery success but also scales more effectively in response to increasing data transmission rates, making it a robust solution for reliable data transfer in dynamic network environments.

**Figure 16:**
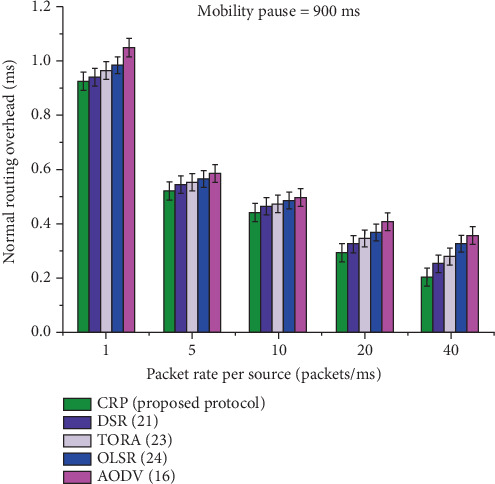
A graphical representation of routing overhead for a node pause time of 900 ms.

Table 12 and Figure 16 present a comparative analysis of RO among five protocols: CRP, DSR, TORA, OLSR, and AODV, at a constant node pause time of 900 ms, evaluated over various data transmission rates. All protocols show a decreasing trend in RO as the rate increases from 1 to 40, reflecting improved performance under heavier traffic. CRP, the proposed protocol, consistently achieves the lowest overhead, while AODV registers the highest values across all rates. The performance gains of CRP over competing protocols range between 5.99% and 8.24%, confirming its superior efficiency in reducing RO under the examined conditions.

**Figure 17:**
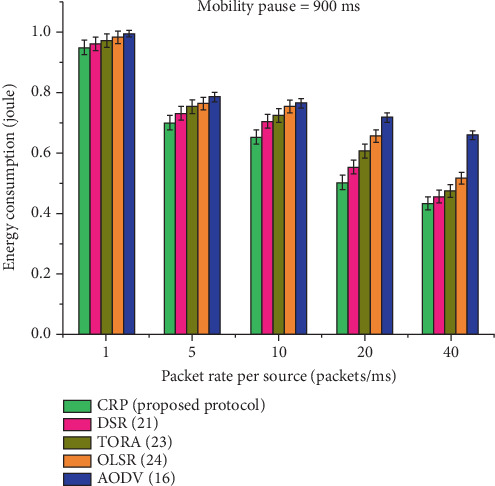
A graphical depiction of energy consumption at a node pause time of 900 ms.

Table 13 and Figure 17 present a comprehensive statistical analysis of energy consumption for five routing protocols: CRP, DSR, TORA, OLSR, and AODV, evaluated at a constant node pause time of 900 ms under varying data transmission rates. A clear downward trend in energy usage is observed across all protocols as the rate increases from 1 to 40, indicating enhanced energy efficiency under higher network loads. Among the compared protocols, CRP consistently achieves the lowest energy consumption, highlighting its superior efficiency. Conversely, AODV records the highest energy usage across all rates. The performance gains of CRP, expressed in energy savings, range from 5.25% to 9.02% over the other protocols, affirming its effectiveness in reducing energy consumption in MANET scenarios.

The results of RO and energy efficiency or normal power consumption (joule) (calculated as per Equation 7) are illustrated in Tables 12 and 13. It appears that CRP consistently outperforms both protocols in the most significant mobility case. In contrast to AODV, TORA, OLSR, and DSR, which had significantly higher overhead, CRP had far lower overhead and was just marginally more expensive (see Figure 16). It is due to an increase in traffic, which does not appear to have had any impact on CRP. The variation in RO is too small, since the CRP handled congestion by anticipating its occurrence and dispersing its adaptive resources over the major and bypass pathways. When the packets were created too quickly, we observed a drop in overhead. This is because there was congestion in the delivery channel in the early case, which led to a low number of control packets being forwarded. At the same time, few nodes participated in the recovery procedure.  Figure 17 reveals that CRP is also the most energy-efficient in steady networks. As additional traffic was added to the network, the advantages of CRPs over the other protocols became more pronounced.

### 4.3. Other Node Mobility Scenario (Pause Time: 300 and 600 ms)

To investigate the impact of different mobility levels, simulations were carried out using pause times of 300 and 600 ms. The resulting performance variations under these conditions are presented in Tables 14, 15, 16, and 17, each corresponding to the respective pause time and performance metric evaluated.

In the first two cases, with pause times set at 0 and 900 ms, CRP proved to be a more efficient and preferred routing protocol than AODV, TORA, OLSR, and DSR, especially under heavy traffic conditions. This pattern is further evident in Tables 14, 15, 16, and 17, where the bolded values highlight instances where CRP achieved a lower average delay than the competing protocols, reinforcing its superior performance. Similarly, for metrics such as PDR, RO, and energy consumption, CRP demonstrates higher PDR and lower RO and energy usage compared to TORA, OLSR, DSR, and AODV. However, these advantages were less frequently observed and primarily occurred under low traffic conditions, where data packets were delivered almost instantaneously and with minimal control effort or energy expenditure.

Across all four experimental scenarios with node pause times of 0, 300, 600, and 900 ms, CRP consistently demonstrated superior performance compared to TORA, OLSR, DSR, and AODV. Specifically, it achieved lower E2E delay, higher PDR, reduced RO, and decreased energy consumption. These performance gains can be attributed to CRP's adaptive routing strategy, congestion-aware features, and energy-efficient design, establishing it as a highly effective protocol for MANETs, particularly under conditions of high traffic.

## 5. Conclusions

The CRP has been introduced to address congestion problems commonly found in MANETs, particularly within routing protocols such as DSR, TORA, OLSR, and AODV. Key performance metrics, including E2E delay, PDR, RO, and energy consumption, have been quantitatively evaluated over a time interval ranging from 0 to 900 ms.

1. As the data rate and transmission timing increased, the CRP values also increased for all the cases. It means that CRP experiences higher packet losses. Moreover, at a data rate of 1 and a timing of 0 ms, the average delay has the minimum CRP value, that is, 0.03, compared to DSR and AODV. Hence, the considered case shows lower packet losses.
2. Similarly, in terms of PDR, at 40 data rates and 600 ms timing, CRP achieves the highest value of 2.34, which is 10.25% greater than DSR and 53.84% greater than AODV, respectively. At 0, 300, and 900 ms data communication timing, the CRP has a higher value than all the others.
3. To reduce the burden on the network, a study of RO was conducted, and it was observed that at 40 data rates and 0 ms timing, the lowest overhead value of 0.23 was achieved compared to all other considered cases. The results show that at 0 ms, communication timing has smooth data transmission compared to the remaining cases using the proposed algorithm.
4. The study on energy consumption has been carried out for data rates 1–40 and communication timings 0–900 ms among CRP, DSR, and AODV, and the results show that it seems that at the 40 rates and 600 ms timing, the CRP obtained 0.41, which is the smallest compared to all the cases. Although a slight deviation was observed with the DSR protocol at 0.40, this is likely due to changes in the machine configuration or operating parameters required for running the algorithm.

This is because CRP seeks to manage congestion rather than resolve it proactively. The bypass idea is crucial to CRP design. The considered ns-3 simulation, which also showed a significant improvement in routing and energy efficiency over AODV, TORA, OLSR, and DSR, confirmed the benefits of CRP.

## Figures and Tables

**Table 1:** Abbreviations listed in the first-level routing table.

**Characteristic**	**Overview**
DST	The destination node
Hop	The next node in the main direction
Hop_status	The next primary hop's level of congestion
Bypass_dst	The place where the bypass route ends
Bypass_hop	The next intersection on the bypass route
Bypass_status	State of the bypass route's congestion
Green_hop	Green is the next node along the main route
Green_metric	Distance in hops to green_hop

**Table 2:** Adjusting splitting probability to adapt to network congestion.

**Congestion**	**Bypass status**		
	**Green**	**Yellow**	**Red**
Next primary node is green	$P≔P+\frac{\left(1-P\right)}{4}$	$P≔P+\frac{\left(1-P\right)}{3}$	$P≔P+\frac{\left(1-P\right)}{2}$
Next primary node is yellow	*P* Unchanged	*P* Unchanged	$P≔P+\frac{\left(1-P\right)}{4}$
Next primary node is red	$P≔P-\frac{\left(1-P\right)}{2}$	$P≔P-\frac{\left(1-P\right)}{4}$	Find Another Bypass

**Table 3:** Simulation parameters for the proposed protocol (CRP).

**Parameters**	**Description**
Total number of nodes	50
Simulation area (m)	1500 × 300
Radio model	Lucent WaveLAN radio interface
Nominal bit rate (Mbps)	2
Threshold, ∈	0.1
Radio range (m)	250
MAC layer	IEEE 802.11DCF (distributed coordination function)
Channel propagation model	Free-space and 2-ray ground reflection model
Routing buffer	64 data packets
Maximum node speed (m/s)	4
Different pause periods (ms)	0, 300, 600, and 900

**Table 4:** When the node pause time is 0 ms, the average E2E delay is observed.

**Rate**	**CRP (proposed protocol)**	**DSR [** 21 **]**	**TORA [** 23 **]**	**OLSR [** 24 **]**	**AODV [** 16 **]**
1	0.030	0.051	0.062	0.073	0.094
5	0.181	0.202	0.213	0.224	0.355
10	0.412	0.423	0.454	0.475	0.556
20	0.253	0.264	0.305	0.356	0.457
40	0.284	0.355	0.456	0.557	0.798
Gain (%)		11.64%	12.68%	13.68%	14.85%

**Table 5:** The E2E uniformity delay at a pause time of 0 ms.

**Rate**	**CRP (proposed protocol)**	**DSR [** 21 **]**	**TORA [** 23 **]**	**OLSR [** 24 **]**	**AODV [** 16 **]**
1	0.021	0.042	0.063	0.074	0.095
5	0.192	0.223	0.254	0.275	0.656
10	0.383	0.414	0.485	0.556	0.757
20	0.404	0.455	0.556	0.607	0.808
40	0.455	0.506	0.607	0.708	0.909
Gain (%)		12.71%	13.65%	15.69%	16.85%

**Table 6:** The statistical PDR data recorded at a pause time of 0 ms.

**Rate**	**CRP (proposed protocol)**	**DSR [** 21 **]**	**TORA [** 23 **]**	**OLSR [** 24 **]**	**AODV [** 16 **]**
1	0.970	0.951	0.902	0.853	0.804
5	1.491	1.452	1.403	1.354	1.305
10	1.652	1.633	1.454	1.405	1.356
20	1.743	1.714	1.655	1.606	1.557
40	1.814	1.785	1.706	1.657	1.608
Gain (%)		1.76%	2.69%	3.68%	4.98%

**Table 7:** Statistical data for routing overhead calculation of different protocols at a node pause time of 0 ms.

**Rate**	**CRP (proposed protocol)**	**DSR [** 21 **]**	**TORA [** 23 **]**	**OLSR [** 24 **]**	**AODV [** 16 **]**
1	1.751	1.802	1.853	1.904	1.955
5	0.622	0.653	0.704	0.755	0.806
10	0.403	0.454	0.505	0.606	0.707
20	0.304	0.375	0.406	0.477	0.578
40	0.255	0.276	0.357	0.408	0.479
Gain (%)		6.75%	7.85%	8.98%	9.78%

**Table 8:** The energy consumption statistics for different protocols when the node pause time is set to 0 ms.

**Rate**	**CRP (proposed protocol)**	**DSR [** 21 **]**	**TORA [** 23 **]**	**OLSR [** 24 **]**	**AODV [** 16 **]**
1	0.702	0.753	0.804	0.855	0.916
5	0.653	0.704	0.755	0.816	0.877
10	0.554	0.615	0.696	0.757	0.808
20	0.515	0.576	0.627	0.668	0.759
40	0.486	0.527	0.558	0.619	0.699
Gain (%)		9.11%	10.52%	11.98%	12.65%

**Table 9:** The statistical data used to calculate the average E2E delay at a node pause time of 900 ms.

**Rate**	**CRP (proposed protocol)**	**DSR [** 21 **]**	**TORA [** 23 **]**	**OLSR [** 24 **]**	**AODV [** 16 **]**
1	1.500	1.601	1.652	1.703	1.784
5	0.301	0.382	0.453	0.654	0.785
10	0.202	0.283	0.384	0.465	0.686
20	0.173	0.214	0.275	0.366	0.467
40	0.124	0.185	0.226	0.277	0.338
Gain (%)		15.87%	16.25%	17.26%	18.95%

**Table 10:** The statistical data for E2E delay uniformity calculation at a node pause time of 900 ms.

**Rate**	**CRP (proposed protocol)**	**DSR [** 21 **]**	**TORA [** 23 **]**	**OLSR [** 24 **]**	**AODV [** 16 **]**
1	0.160	0.182	0.190	0.202	0.223
5	0.151	0.162	0.171	0.183	0.204
10	0.140	0.153	0.162	0.174	0.185
20	0.130	0.140	0.153	0.165	0.176
40	0.122	0.130	0.144	0.156	0.167
Gain (%)		9.10%	11.22%	12.33%	14.99%

**Table 11:** The statistical data used to calculate PDR when the node pause time is 900 ms.

**Rate**	**CRP (proposed protocol)**	**DSR [** 21 **]**	**TORA [** 23 **]**	**OLSR [** 24 **]**	**AODV [** 16 **]**
1	0.980	0.951	0.942	0.933	0.924
5	1.311	1.292	1.253	1.204	1.185
10	1.632	1.583	1.504	1.455	1.356
20	2.253	2.204	2.185	2.166	2.107
40	2.314	2.295	2.256	2.207	2.158
Gain (%)		1.94%	2.93%	3.98%	4.69%

**Table 12:** At 900 ms pause time, routing overhead is reflected in the data.

**Rate**	**CRP (proposed protocol)**	**DSR [** 21 **]**	**TORA [** 23 **]**	**OLSR [** 24 **]**	**AODV [** 16 **]**
1	0.925	0.940	0.965	0.985	1.050
5	0.521	0.543	0.554	0.565	0.586
10	0.442	0.464	0.475	0.486	0.497
20	0.293	0.325	0.346	0.367	0.408
40	0.204	0.256	0.277	0.328	0.359
Gain (%)		5.99%	6.52%	7.25%	8.24%

**Table 13:** Energy consumption is recorded at 900 ms pause time.

**Rate**	**CRP (proposed protocol)**	**DSR [** 21 **]**	**TORA [** 23 **]**	**OLSR [** 24 **]**	**AODV [** 16 **]**
1	0.950	0.962	0.973	0.984	0.995
5	0.701	0.733	0.754	0.765	0.786
10	0.652	0.704	0.725	0.756	0.767
20	0.503	0.555	0.606	0.657	0.718
40	0.434	0.456	0.477	0.518	0.659
Gain (%)		5.25%	6.30%	7.85%	9.02%

**Table 14:** The CRP illustrations show better E2E delay and PDR than others at 300 ms pause.

	**Average E2E delay**					**PDR**				
**Rate**	**CRP (proposed protocol)**	**DSR [** 21 **]**	**TORA [** 23 **]**	**OLSR [** 24 **]**	**AODV [** 16 **]**	**CRP (proposed protocol)**	**DSR [** 21 **]**	**TORA [** 23 **]**	**OLSR [** 24 **]**	**AODV [** 16 **]**
1	0.980	1.051	1.152	1.203	1.354	0.970	0.901	0.882	0.863	0.804
5	0.201	0.282	0.383	0.584	0.765	0.991	0.882	0.773	0.664	0.505
10	0.252	0.303	0.354	0.485	0.656	1.652	1.503	1.454	1.405	1.306
20	0.283	0.324	0.345	0.366	0.607	1.883	1.774	1.675	1.556	1.257
40	0.204	0.255	0.326	0.337	0.588	1.954	1.905	1.856	1.817	1.788
Gain (%)		15.36%	16.33%	18.33%	20.33%		6.51%	7.85%	10.66%	12.88%

**Table 15:** At 300 ms pause, CRP improves routing overhead and energy efficiency over DSR, TORA, OLSR, and AODV.

	**Routing overhead**					**Energy**				
**Rate**	**CRP (proposed protocol)**	**DSR [** 21 **]**	**TORA [** 23 **]**	**OLSR [** 24 **]**	**AODV [** 16 **]**	**CRP (proposed protocol)**	**DSR [** 21 **]**	**TORA [** 23 **]**	**OLSR [** 24 **]**	**AODV [** 16 **]**
1	2.600	2.801	2.852	2.903	2.984	0.970	0.981	0.992	1.053	1.104
5	0.441	0.602	0.653	0.754	0.905	0.951	1.052	1.103	1.194	1.295
10	0.302	0.353	0.454	0.585	0.656	0.552	0.653	0.704	0.875	0.906
20	0.263	0.304	0.385	0.486	0.607	0.523	0.604	0.655	0.706	0.857
40	0.204	0.285	0.356	0.457	0.508	0.504	0.555	0.606	0.657	0.818
Gain (%)		14.04%	16.29%	18.98%	21.99%		9.86%	11.68%	13.88%	15.89%

**Table 16:** At a 600 ms pause, CRP is more efficient than DSR, TORA, OLSR, and AODV in terms of E2E delay and PDR.

	**Average E2E delay**					**PDR**				
**Rate**	**CRP (proposed protocol)**	**DSR [** 21 **]**	**TORA [** 23 **]**	**OLSR [** 24 **]**	**AODV [** 16 **]**	**CRP (proposed protocol)**	**DSR [** 21 **]**	**TORA [** 23 **]**	**OLSR [** 24 **]**	**AODV [** 16 **]**
1	0.970	0.991	1.052	1.253	1.354	0.980	0.881	0.852	0.803	0.754
5	0.801	0.852	0.903	0.934	0.995	1.251	1.152	1.103	1.054	1.015
10	0.602	0.653	0.724	0.825	0.906	1.752	1.503	1.454	1.405	1.106
20	0.553	0.604	0.655	0.716	0.837	2.103	2.054	1.955	1.786	1.707
40	0.434	0.485	0.556	0.657	0.718	2.344	2.105	2.056	1.957	1.808
Gain (%)		6.70%	8.29%	10.89%	12.89%		8.72%	9.85%	10.88%	12.88%

**Table 17:** The improvement in CRP performance over DSR, TORA, OLSR, and AODV in terms of routing overhead and energy efficiency at a pause period of 600 ms.

	**Routing overhead**					**Energy**				
**Rate**	**CRP (proposed protocol)**	**DSR [** 21 **]**	**TORA [** 23 **]**	**OLSR [** 24 **]**	**AODV [** 16 **]**	**CRP (proposed protocol)**	**DSR [** 21 **]**	**TORA [** 23 **]**	**OLSR [** 24 **]**	**AODV [** 16 **]**
1	4.40	4.55	4.60	4.65	4.80	0.961	0.992	1.053	1.104	1.155
5	0.45	0.65	0.70	0.75	0.83	0.762	0.883	0.914	0.945	0.996
10	0.29	0.35	0.45	0.55	0.65	0.553	0.664	0.705	0.736	0.807
20	0.24	0.39	0.43	0.50	0.61	0.484	0.585	0.656	0.707	0.758
40	0.23	0.30	0.36	0.45	0.58	0.415	0.506	0.617	0.658	0.709
Gain (%)		11.23%	13.52%	15.38%	17.88%		14.33%	15.87%	16.85%	18.99%

## Data Availability

All the data used to support the findings of this study are included within the article.

## Nomenclature

AODV: Ad hoc On-Demand Distance Vector
CRP: congestion-adaptive routing protocol
DSR: dynamic source routing protocol
MANETs: mobile ad hoc networks
MAC: media access control
ms: millisecond
NS: network simulator
NCAM: new congestion avoidance method
OLSR: Optimized Link State Routing
REQ: packet request
REP: packet reply
Sec: second
TCP: transmission control protocol
TTL: time to live
TORA: Temporally Ordered Routing Algorithm
